# Common Cold Coronavirus 229E Induces Higher Interferon Stimulating Gene Responses in Human Nasal Epithelial Cells from Patients with Chronic Rhinosinusitis with Polyposis

**DOI:** 10.1177/19458924241276274

**Published:** 2024-11-08

**Authors:** Elizabeth A. Sell, Li Hui Tan, David M. Renner, Jennifer Douglas, Robert J. Lee, Michael A. Kohanski, John V. Bosso, David W. Kennedy, James N. Palmer, Nithin D. Adappa, Susan R. Weiss, Noam A. Cohen

**Affiliations:** 1 6569 Perelman School of Medicine at the University of Pennsylvania, Philadelphia, PA, USA; 2 Division of Rhinology, Department of Otorhinolaryngology–Head and Neck Surgery, Perelman School of Medicine, 6572 University of Pennsylvania, Philadelphia, PA, USA; 3 Department of Microbiology, University of Pennsylvania, Philadelphia, PA, USA; 4 Department of Microbiology, Perelman School of Medicine, 6572 University of Pennsylvania, Philadelphia, PA, USA; 5 Corporal Michael J. Crescenz Veterans Administration Medical Center, Philadelphia, PA, USA; 6 10630 Monell Chemical Senses Center, Philadelphia, PA, USA

**Keywords:** common cold, 229E, coronavirus, rhinosinusitis, chronic rhinosinusitis, rhinovirus, interferon, type 1 inflammation, nasal polyps, virus

## Abstract

**Background:**

Viral infections have long been implicated in the development of chronic rhinosinusitis with nasal polyps (CRSwNP). Given widespread exposure to the common cold coronavirus 229E (HCoV229E), we sought to investigate how HCoV-229E is cleared and stimulates interferon pathways in air–liquid interface (ALI) cultures from patients with CRSwNP.

**Objective:**

The objective of this study was to identify whether viral clearance and ISG expression is different in ALI cultures from donors with CRSwNP compared with controls.

**Methods:**

Plaque assays were used to quantify infectious virus released by infected air–liquid interface (ALI) cultures derived from patients with CRSwNP compared to patients without CRS (controls). Additionally, mock and induced levels of Interferon Stimulated Genes (ISGs) mRNA following HCoV-229E infection were quantified by RT-qPCR.

**Results:**

Quantification of infectious virus by plaque assay reveals that CRSwNP ALI cultures were equally susceptible to HCoV-229E infection, and surprisingly viral titers dropped significantly faster than in the control ALI cultures. We further demonstrate that this accelerated viral clearance correlates with increased mRNA expression of at least 4 ISGs following viral infection in the CRSwNP ALIs compared to the control ALIs.

**Conclusion:**

This study paradoxically demonstrates that ALI cultures from patients with CRSwNP are more efficient at clearing the common cold HCoV-229E virus compared to controls. We also demonstrate significantly increased ISG mRNA expression following HCoV-229E infection in CRSwNP. These findings call for further investigation into the effect of unimpaired interferon signaling on the type 2 inflammatory environment in patients with CRSwNP.

## Introduction

Inflammatory diseases of the respiratory system – including chronic rhinosinusitis (CRS) in the upper airway and asthma in the lower airway – are multifactorial diseases involving increased persistent inflammation of the sinonasal or bronchial mucosa. Patients with CRS with nasal polyps (CRSwNP) characterized by a type 2 immune profile often have severe disease, and asthma is often a comorbidity in these patients. Both disease states are characterized by tissue eosinophilia and high local IgE levels.^1^ Viral infections of the upper airway in early life have been associated with an increased risk of developing asthma later in life.^2,3,4^ Higher incidences of viruses have been reported from nasopharyngeal clinical swabs during asthma attacks in adults.^5,6^ Furthermore, patients with asthma demonstrate increased viral replication and persistent higher viral titers both *in vitro* and in bronchioalveolar lavage fluid, which correlates with decreased induction of type I and type III interferons.^7^ Overall, there is strong evidence supporting the role of viral infections in both the development of asthma and in asthma exacerbations; however, the role of viral infections in the pathogenesis of CRSwNP is much less well studied.

**Figure 1.**
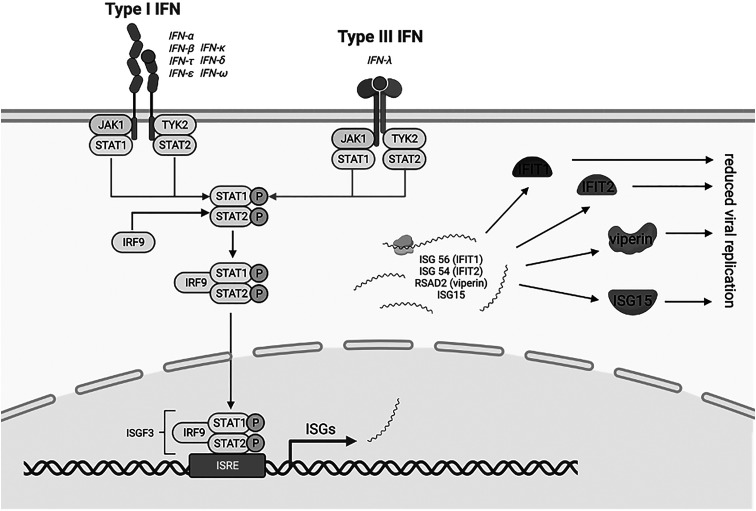
Interferon and activation of classical JAK-STAT pathways by type I and III interferons. The type I and III IFN receptors are both associated with the Janus activated kinase 1 (JAK1). Activation of JAK1 results in tyrosine phosphorylation of STAT1 (signal transducer and activator of transcription) and STAT2, which leads to the formation of complexes that translocate to the nucleus and bind IFN-stimulated response elements (ISREs) in DNA to initiate gene transcription. Gene transcription then produces mRNA of interferon-stimulating genes (ISGs), including ISG56 (IFIT1), ISG54 (IFIT2), Viperin (gene name RSAD2), and ISG15. While functionally distinct, these ISGs collaborate to reduce viral replication. Created using BioRender (biorender.com; BioRender, Toronto, Ontario, Canada).

Viral infections are thought to be possible contributors to both the initiation and progression of CRSwNP.^8,9,10^ Human rhinovirus 16 (HRV16) infection has been shown to result in mucous hypersecretion, alterations to tight junctions, and increased production of remodeling factors in CRSwNP.^11,12,13^ Additionally, researchers have found that respiratory viruses were more commonly isolated from patients with CRS compared with controls, with common cold coronaviruses being the most commonly isolated.^14,15^ Mechanistically, this could be due to differences in interferon production, given that interferon in human airway epithelial cells is essential for viral clearance.^16,17^ In response to viral infection, epithelial cells in the respiratory tract produce antiviral factors that include interferons, which contribute to clearance of the virus (Figure 1).^18,19,20,21^ Interferon signaling is mediated by many interferon-stimulating genes (ISGs), which inhibit viral replication.^22^ There is evidence that baseline interferon levels are decreased in patients with CRSwNP due to the type 2 inflammatory environment.^23,24,25^ Given the lower basal levels, it was hypothesized that interferon production is also deficient during viral infection in patients with CRSwNP, which would result in prolonged infection and delayed viral clearance, similar to the role that viral infections are thought to play in the pathogenesis of asthma. Furthermore, a possible explanation, at least in part, for the rising incidence of asthma and CRSwNP in industrialized countries in the last few decades is epigenetic changes secondary to environmental exposures.^26,27^ The role of epigenetics, including DNA methylation and posttranslational histone modifications, has been increasingly investigated in immune mechanisms underlying asthma and CRSwNP, highlighting the importance of investigating epithelial responses to viral infections.

The average healthy adult experiences between 1 and 3 common colds per year, which are caused by rhinoviruses (30%–50%) and coronaviruses including strains 229E, OC43, NL63, and HKU1 (5%–30%), as well as respiratory syncytial viruses (RSV) and adenoviruses, among others.^28^ One study found that greater than 90% of over 100 patients from seven U.S. cities were seropositive for four coronavirus strains, with 99% having antibodies to human coronavirus 229E.^29^ Given the evidence for widespread exposure to coronavirus 229E, we sought to investigate how the common cold coronavirus 229E affects interferon pathways in air–liquid interface (ALI) cultures derived from patients with CRSwNP. This was accomplished by infecting cultures with 229E and measuring infectious virus by plaque assay at different time points following infection. Furthermore, we also evaluated ISG mRNA expression following viral infection in cultures compared with donors with CRSwNP compared with controls.

## Materials and Methods

*Patient demographics/clinical descriptive data analysis:* Patient demographics and comorbid conditions were analyzed in Prism (Graphpad, San Diego, CA). Mean and standard deviation were determined for age and differences between the CRSwNP and control groups were determined by t-test. Differences in sex and race between the CRSwNP and control groups were determined using chi-square test.

*Reagents and experimental solutions:* Cell culture reagents (DMEM, amino acids, antibiotics, etc) and Dulbecco's PBS reagents were obtained as previously described.^30^

*Tissue acquisition:* Tissue samples were obtained from patients (Table 1) recruited from the Department of Otorhinolaryngology – Head and Neck Surgery, Division of Rhinology, University of Pennsylvania and the Philadelphia Veterans Affairs Medical Center after obtaining IRB approval and written consent from all patients involved. Patients with a history of systemic inheritable disease (*eg* cystic fibrosis, granulomatosis with polyangiitis, systemic immunodeficiencies) or immunosuppressive medications were excluded from the study. For this study, patients with a history of asthma or allergic rhinitis were also excluded. Epithelial brushings were obtained under direct visualization using at least two cytology brushes rotated against the epithelium of the middle meatus, as identified by the attending otolaryngologist. The cytology brushes were then placed on ice immediately following collection in 15 mL conical tubes containing 10 mL sterile saline and transported to the laboratory.

*Generation of pooled primary sinonasal ALI cultures:* Sinonasal mucosal specimens were acquired and ALI cultures were established from enzymatically dissociated human sinonasal epithelial cells (HSECs) as previously described.^30,31,32^ The cells were pooled in a 1:1:1 or 1:1:1:1:1 ratio after basal cell proliferation. The cultures were allowed to differentiate for 3 weeks in differentiation media prior to infection. Confirmatory tests for differentiation were performed on ALI cultures prior to infection, including epithelial morphology assessed via light microscopy to confirm ciliation.

*Virus*: Human coronavirus 229E was propagated in Huh7 cells, a human immortalized well-differentiated hepatocyte-derived carcinoma cell line. Low MOI (0.01) infections were used to generate the viral stock for all experiments. The viral stock was sequenced and found to be comparable to wild-type reference sequences available on the National Center for Biotechnology Information (NCBI).

*Infections and Plaque Assay*: All infections were conducted at MOI = 5 plaque forming units (PFU)/cell. Viruses were diluted in serum-free Dulbecco's modified Eagle's medium (DMEM) to achieve a total inoculum volume of 50μL, which was added apically to the nasal ALI cultures. Virus was allowed to adsorb to the nasal ALI cultures for an incubation period of 1 h at 33 °C.^33^ The virus was then aspirated and the nasal ALI cultures were washed three times with phosphate-buffered saline (PBS). ALI cultures were then incubated at 33 °C following infection. At the indicated time points in each experiment, 200μL PBS was added to the apical surface of each previously infected transwell, collected and then frozen at −80 °C. For plaque assays, Huh7 cells were plated in 6-well plates. Viral apical washes were diluted in serum-free DMEM and added to plates for adsorption for 1 h at 33 °C. After adsorption, 4 mL liquid overlay (1x sodium pyruvate, 2% FBS, 0.1% agarose) was added to each well. Plates were then incubated at 33 °C. Cells were fixed using 4% paraformaldehyde at 3 days post-infection and virus plaques were visualized by crystal violet staining.

*RNA Extraction and Quantitative RT-PCR Analysis*: Cells were lysed with RLT Plus buffer (Qiagen RNeasy Plus kit) and RNA was extracted following the Qiagen protocol. RNA was reverse transcribed into complementary DNA (cDNA) using the Applied Biosystems High Capacity Reverse Transcriptase Kit. cDNA was amplified using specific qRT-PCR primers for each target gene using Bio-Rad iQ SYBR Green Supermix and the Thermo Fisher QuantStudio 3 PCR system. Primer sequences are shown in Supplemental Table 1. Δ C_T_ values were calculated using the following formula: Δ C_T _= C_T target gene_ – C_T 18S_. Technical triplicates were averaged and changes in mRNA levels were reported as fold changes over sham-treated cultures (50μL DMEM with no virus incubated for 1 h and then washed 3 times with PBS), using the formula $2^{-\Delta (\Delta Ct)}$ .

*Data Analyses:* Plotting of data and statistical analysis were performed using GraphPad Prism software (GraphPad Software, Inc.). Statistical significance was assessed by comparing one-way ANOVA. Displayed significance is determined by P value, where * = P < 0.05; ** = P < 0.01; *** = P < 0.001; and **** = P < 0.0001; ns = not significant, and, in some figures, ns is not displayed on the graph.

**Table 1.** Demographic characteristics of CRSwNP and control patients.

		CRSwNP (n = 8)	Control (n = 8)	p-value
Sex	Male	5	7	0.20
	Female	3	1	
Age		42.5 (13.8)	43.5 (15.6)	0.85
Race	White	6	5	0.67
	African American	2	2	
	Hispanic	0	1	

## Results

**Figure 2.**
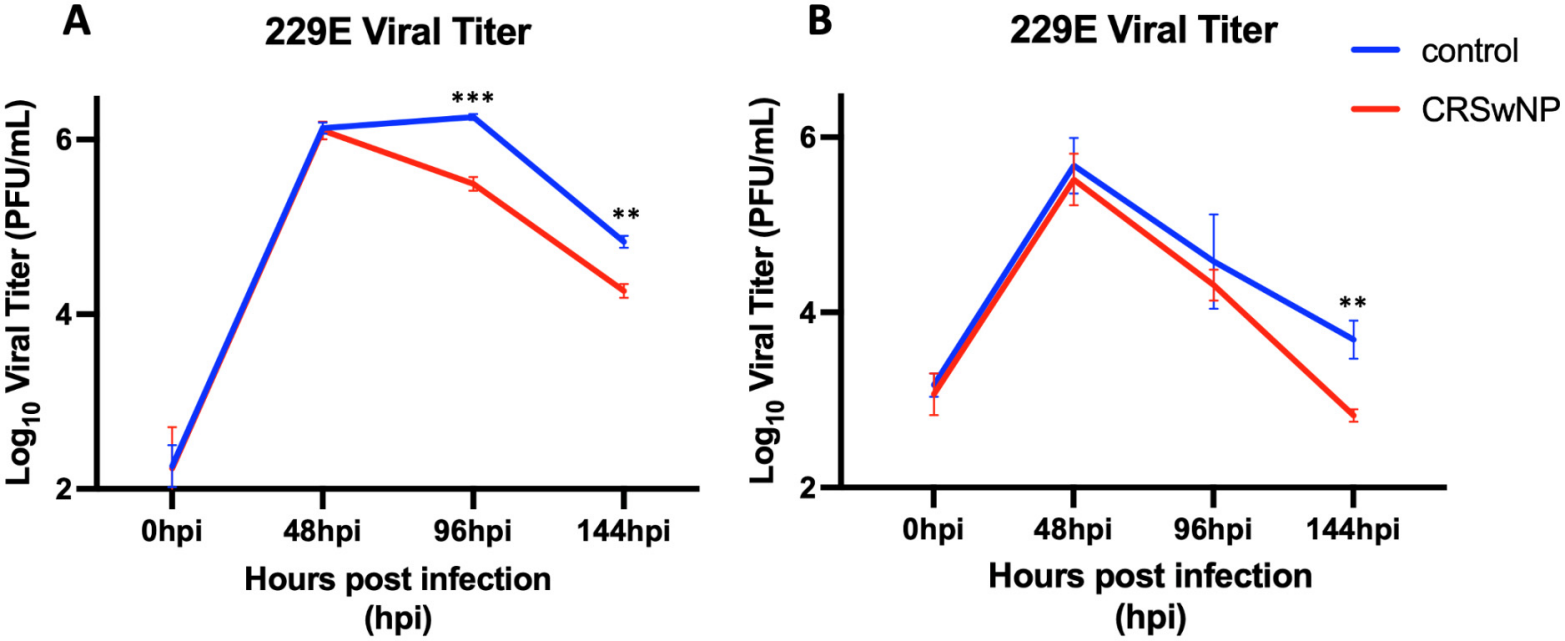
Common cold coronavirus 229E clears faster in ALI cultures from patients with CRSwNP. ALI pooled cultures from 3 control donors without CRS and 3 donors with CRSwNP were infected with 229E (MOI = 5) in triplicate ALI cultures and viral titers were measured at 48-, 96- and 144-hours post infection (hpi) (A). In a second infection, ALI pooled cultures from 5 different control donors without CRS and 5 different donors with CRSwNP were infected with 229E (MOI = 5) again in triplicate ALI cultures (B). * = P < 0.05; ** = P < 0.01; *** = P < 0.001.

To investigate the effect of the common cold human coronavirus 229E on the upper airway innate immune response, we infected primary human sinonasal ALI cultures with 229E virus and assessed infectious virus at various timepoints following infection by plaque assay. We first generated pooled ALI cultures from three control donors without CRS and three donors with CRSwNP and measured infectious titers at 48-, 96-, and 144-h post infection (hpi). We found that the pooled ALI cultures from the donors with CRSwNP were not only equally susceptible to 229E infection but cleared the virus significantly faster than the non-CRS control ALI cultures at both 96- and 144-hpi (Figure 2A). To confirm this observation, we generated additional pooled ALI cultures from five different control donors without CRS and five different donors with CRSwNP and again measured infectious titers at 48-, 96- and 144-hpi. Again, we found that the pooled ALI cultures from the five donors with CRSwNP cleared the virus faster than the pooled ALI cultures from the five donors without CRS (Figure 2B). These data demonstrate that in an *in vitro* model, sinonasal epithelial cultures derived from patients with CRSwNP are more efficient at clearing the 229E virus compared to cultures derived from patients without CRS.

**Figure 3.**
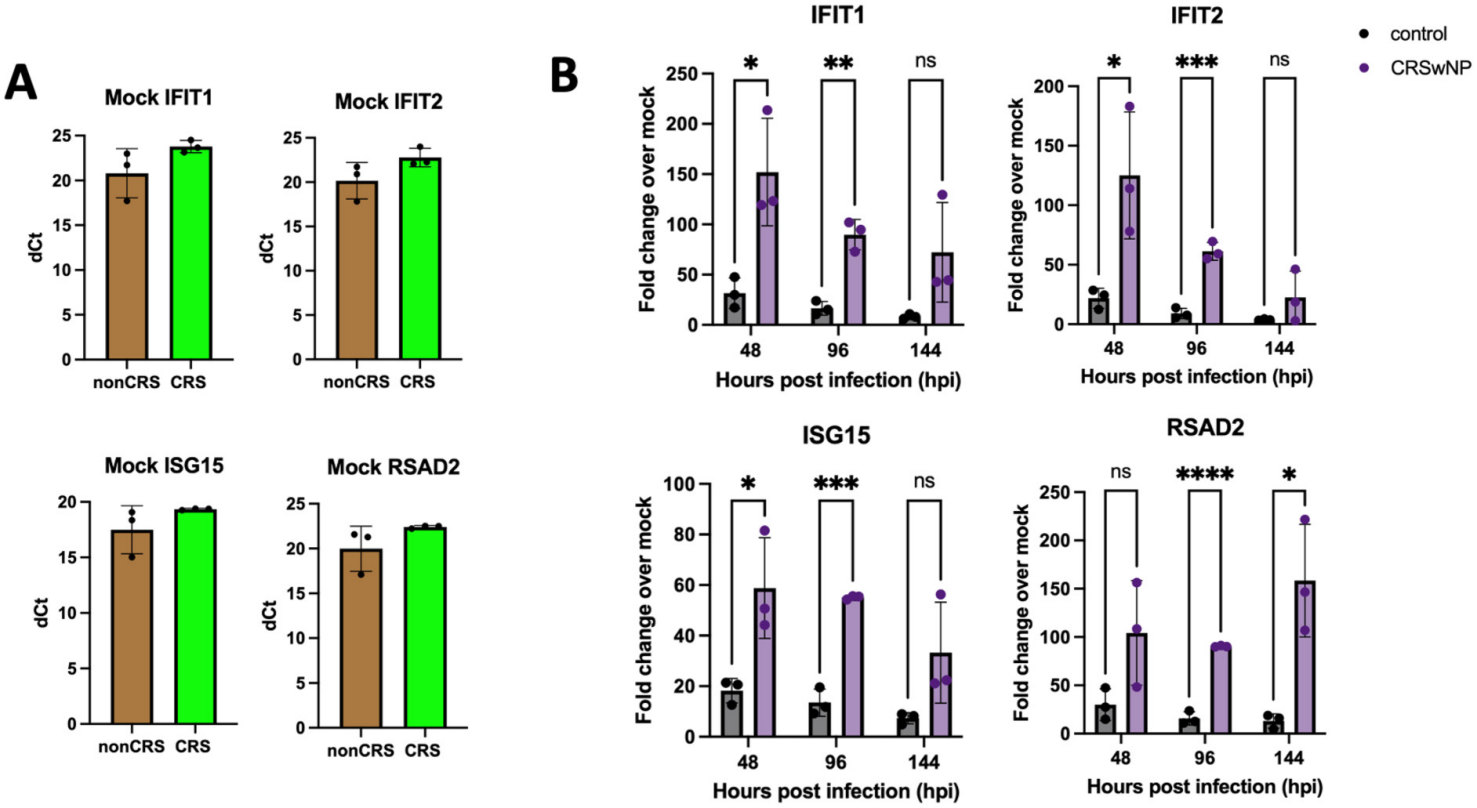
Common cold coronavirus 229E increases ISG mRNA expression significantly more in ALI cultures from patients with CRSwNP by RT-qPCR. Fold changes were calculated by subtracting the measured fold change of the infected ALIs from the averaged mock values for nonCRS and CRSnp, respectively (A). ALI pooled cultures from 5 control donors without CRS and 5 donors with CRSwNP were infected with 229E (MOI = 5) in triplicate ALI cultures and ISG mRNA fold changes were measured at 48-, 96- and 144-hours post infection (hpi) (B). * = P < 0.05; ** = P < 0.01; *** = P < 0.001; and **** = P < 0.0001; ns = not significant.

Given that the production of interferon is known to play a crucial role in viral clearance, we next sought to measure ISG responses to a series of canonical antiviral genes in the pooled ALI cultures from patients without CRS compared to the pooled ALI cultures from patients with CRSwNP. First, mock levels of ISG mRNA in uninfected cultures were quantified by RT-qPCR. We found that none of the ISG mRNA under investigation demonstrated differential basal expression between the pooled ALI cultures from donors with or without CRSwNP (Figure 3A). Although not statistically significant, we did find that average ΔC_T_ values were higher for ISGs including IFIT1, IFIT2, RSAD2 (the ISG that encodes the protein viperin), and ISG15 in cultures from patients with CRSwNP, suggesting lower basal levels and consistent with previous reports demonstrating lower levels of interferon and ISGs in cultures from patients with CRSwNP.^23,34^ However, we wanted to further investigate what happens to ISG levels relative to these respective mock values when the cultures were challenged with a viral infection.

We infected the pooled ALI cultures with 229E in triplicate and found that the pooled ALI cultures from donors with CRSwNP exhibited significantly higher ISG mRNA expression across four ISGs as quantified by RT-qPCR (Figure 3B). We found that mRNA expression of IFIT1, IFIT2, and ISG15 peaked at 48-hpi and trended down at later timepoints (96- and 144-hpi), while mRNA expression of RSAD2 (viperin) remained elevated throughout all timepoints measured (48-, 96-, and 144-hpi). This finding suggests that viral replication and clearance have different effects on different ISG mRNA in the context of 229E infection. Overall, our results demonstrate that sinonasal epithelium derived from patients with CRSwNP is paradoxically more efficient at clearing 229E compared to sinonasal epithelium derived from non-CRS control patients. This faster viral clearance correlates with increased ISG mRNA expression.

## Discussion

In this study, we demonstrate that ALI cultures derived from patients with CRSwNP are more efficient at clearing the human common cold coronavirus 229E compared to ALI cultures derived from controls. Quantification of infectious virus by plaque assay demonstrates that ALI cultures generated from patients with CRSwNP show decreasing titers faster than in ALI cultures originating from patients without CRS in two separate infections from different donors (n = 8 for each cohort). We also demonstrate significantly increased mRNA copy number in nasal epithelium derived from CRSwNP compared to controls across four ISGs as quantified by RT-qPCR after 229E infection. Taken together, this study supports previous work demonstrating that patients with CRSwNP do not exhibit delayed viral clearance or impaired interferon signaling. This warrants further investigation into how interferon signaling interacts with the type 2 inflammatory environment classically found in patients with CRSwNP.

Type I interferon responses have been linked to type 2 inflammation and antiviral functions in the sinonasal epithelium. Jang *et al* reported findings suggesting that type I interferon can contribute to eosinophilic CRS and the type 2 inflammatory environment, specifically to the production of IL-5 and IL-13.^35^ Additionally, Ko and colleagues demonstrated that HRV16 possibly increases IL-4 production in ALI cultures from some subtypes of patients with CRS.^36^ In whole-transcriptome RNA-sequencing studies, type 1 interferon signaling has been noted to be enriched in patients with CRSwNP.^37^ Additionally, type III interferon has been found to be a marker in patients with CRSwNP undergoing revision surgery,^38^ although subsequent studies have not corroborated this finding.^39^ In addition to its role in viral clearance, interferon-γ (IFN-γ) is a type III interferon that has been implicated in fungal eosinophilic rhinosinusitis.^40,41^ Furthermore, high type III interferon levels have been described in patients with aspirin-exacerbated respiratory disease (AERD), which has also been shown to increase the expression of genes involved in leukotriene synthesis.^42^ Overall, our results are consistent with previous findings that interferon signaling is not impaired in patients with CRSwNP unlike in patients with asthma.^23,34^

Evidence suggests that interferon signaling is not delayed in patients with CRSwNP as it is in patients with asthma.^43,44,45^ and given that the interferon response may be active to a greater degree in patients with CRSwNP compared with patients with asthma, it is important to investigate interferon signaling in the context of the chronic inflammation in CRSwNP. No other studies to date have looked at other common cold viruses such as human common cold coronaviruses in the context of CRSwNP. This work demonstrates the need for future investigations into how common cold viruses affect interferon signaling and inflammation in the sinonasal epithelium, particularly how they could interact with mediators of type 2 inflammation. Further work into how interferon signaling interacts with the type 2 inflammatory environment in patients with CRSwNP could yield insights into the mechanisms underlying the development of chronic inflammation in these patients.

## Conclusion

In summary, this study demonstrates that sinonasal ALI cultures generated from patients with CRSwNP are more efficient at clearing the human common cold coronavirus 229E compared to ALIs derived from non-CRS control patients. We also demonstrate significantly increased mRNA copy number of ISGs as quantified by RT-qPCR in cultures from patients with CRSwNP. A greater understanding of the effects of interferon signaling could reveal important insights into the interplay between interferon signaling and type 2 inflammation in the development of chronic inflammation in patients with CRSwNP.

## Supplemental Material

sj-docx-1-ajr-10.1177_19458924241276274 - Supplemental material for Common Cold Coronavirus 229E Induces Higher Interferon Stimulating Gene Responses in Human Nasal Epithelial Cells from Patients with Chronic Rhinosinusitis with Polyposis Supplemental material, sj-docx-1-ajr-10.1177_19458924241276274 for Common Cold Coronavirus 229E Induces Higher Interferon Stimulating Gene Responses in Human Nasal Epithelial Cells from Patients with Chronic Rhinosinusitis with Polyposis by Elizabeth A. Sell, B.A. , Li Hui Tan, PhD, David M. Renner, PhD, Jennifer Douglas, M.D., Robert J. Lee, PhD, Michael A. Kohanski, M.D. PhD, John V. Bosso, M.D., David W. Kennedy, M.D., James N. Palmer, M.D., Nithin D. Adappa, M.D., Susan R. Weiss, PhD, and Noam A. Cohen, M.D. PhD in American Journal of Rhinology & Allergy
