# Supplementary material for: Common Cold Coronavirus 229E Induces Higher Interferon Stimulating Gene Responses in Human Nasal Epithelial Cells from Patients with Chronic Rhinosinusitis with Polyposis
Source: Am J Rhinol Allergy. 2024 Nov 8;39(1):13–20. doi: 10.1177/19458924241276274 (PMC11626848; doi:10.1177/19458924241276274)
Supplement: sj-docx-1-ajr-10.1177_19458924241276274 - Supplemental material for Common Cold Coronavirus 229E Induces Higher Interferon Stimulating Gene Responses in Human Nasal Epithelial Cells from Patients with Chronic Rhinosinusitis with Polyposis [file sj-docx-1-ajr-10.1177_19458924241276274.docx]

**Table 1 Primers**

| **Primer** | **Sequence** |
| --- | --- |
| 18S FW | TTCGATGGTAGTCGCTGTGC |
| 18S REV | CTGCTGCCTTCCTTGAATCTGGTA |
| hIFIT1 FW | TGGTGACCTGGGGCAACTTT |
| hIFIT1 REV | AGGCCTTGGCCCGTTCATAA |
| hIFIT2 FW | CTGAGAATTGCACTGCAACCATG |
| hIFIT2 REV | GTAGGCTGCTCTCCAAGGAATTC |
| RSAD2 FW | CACAAAGAAGTGTCCTGCTTGGT |
| RSAD2 REV | AAGCGCATATATTCATCCAGAATAAG |
| ISG15 FW | CATCTTTGCCAGTACAGGAGC |
| ISG15 REV | GGGACACCTGGAATTCGTTG |
